# Large SARS-CoV-2 Outbreak Caused by Asymptomatic Traveler, China

**DOI:** 10.3201/eid2612.203437

**Published:** 2020-12

**Authors:** Andrei R. Akhmetzhanov

**Affiliations:** College of Public Health, National Taiwan University, Taipei, Taiwan

**Keywords:** respiratory infections, severe acute respiratory syndrome coronavirus 2, SARS-CoV-2, SARS, COVID-19, coronavirus disease, zoonoses, viruses, coronavirus, contact tracing, isolation

**To the Editor:** Liu et al. (*1*) reported on a large outbreak of >70 cases of severe acute respiratory syndrome coronavirus 2 (SARS-CoV-2) infection. The origin of the outbreak was traced back to an asymptomatically infected traveler. However, delays in detecting SARS-CoV-2 infections in families B and C1 represent missed opportunities for earlier isolation and interruption of disease transmission.

After reading Lui et al. (*1*), we questioned whether April 7 was the first day of illness onset for the initial confirmed case, B2.3. Because viral load and infectiousness peak around the time of symptom onset and exposure of family C1 to case B2.3 was 9 days before that date, presymptomatic transmission would be highly unlikely (*2*). Although B2.2, who was an asymptomatic carrier, also could have played a role in exposing the family of C1, a close examination of publicly available records (*3*) altered this hypothesis.

Exposed on March 26, case B2.3 transmitted the virus to family C1 3 days later, on March 29, which appears to be 1 day before his first symptoms. Case B2.3 went to an outpatient clinic with a subjective fever on March 30 but was not tested for SARS-CoV-2. He was not isolated until he went to a clinic again on April 7 with worsening symptoms. Earlier isolation and testing of B2.3 could have prompted earlier contact tracing and triggered earlier diagnosis of C1 during his hospital stay, potentially preventing the chain of >60 SARS-CoV-2 transmissions in 2 hospitals.

The uncooperative behavior of cases B2.2 and B2.3 complicated efforts for early contact tracing (*3*), demonstrating cooperation with medical officers, coupled with proactive case-finding and earlier case isolation, clearly are crucial in curbing disease spread (*4*,*5*). If timely actions had been implemented, the outbreak could have been prevented or greatly reduced in size.
